# The Role of Endocrine G Protein-Coupled Receptors in Ovarian Cancer Progression

**DOI:** 10.3389/fendo.2017.00066

**Published:** 2017-04-07

**Authors:** Qingyu Zhang, Nadine Ellen Madden, Alice Sze Tsai Wong, Billy Kwok Chong Chow, Leo Tsz On Lee

**Affiliations:** ^1^Centre of Reproduction, Development and Aging, Faculty of Health Sciences, University of Macau, Taipa, Macau; ^2^School of Biological Sciences, The University of Hong Kong, Pokfulam, Hong Kong

**Keywords:** G protein-coupled receptor, ovarian cancer, endocrine system, peptide hormone, cell signaling

## Abstract

Ovarian cancer is the seventh most common cancer in women and the most lethal gynecological cancer, causing over 151,000 deaths worldwide each year. Dysregulated production of endocrine hormones, known to have pluripotent effects on cell function through the activation of receptor signaling pathways, is believed to be a high-risk factor for ovarian cancer. An increasing body of evidence suggests that endocrine G protein-coupled receptors (GPCRs) are involved in the progression and metastasis of ovarian neoplasms. GPCRs are attractive drug targets because their activities are regulated by more than 25% of all drugs approved by the Food and Drug Administration. Therefore, understanding the role of endocrine GPCRs during ovarian cancer progression and metastasis will allow for the development of novel strategies to design effective chemotherapeutic drugs against malignant ovarian tumors. In this review, we address the signaling pathways and functional roles of several key endocrine GPCRs that are related to the cause, progression, and metastasis of ovarian cancer.

## G Protein-Coupled Receptors (GPCRs) and Ovarian Cancer

According to the 2012 global cancer statistical report from the World Health Organization (WHO) BLOBOCAN project, approximately 200,000 new cases of ovarian cancer are reported annually (1–3). It is the second most common gynecological cancer and is associated with the higher mortality rate of any gynecological cancer. The high mortality rate is due to the aggressive but asymptomatic progression of cancer cells throughout the peritoneal cavity, with more than 70% of patients being diagnosed at an advanced/metastatic stage (stage III or IV) (4). Because ovarian cancers are frequently diagnosed late, most patients present with extensive intraperitoneal tumors. Surgical debulking followed by chemotherapy is the mainstay of treatment for ovarian cancer, but it has not proven to be effective. The 5-year survival rate of patients undergoing salvage therapies is less than 25%. More than 70% of patients are initially sensitive to platinum- and taxane-based chemotherapy, but recurrence and peritoneal metastases are found in more than half of these patients, leading to low overall survival rates (5). The WHO classifies ovarian cancer into epithelial, sex cord-stromal, and germ cell neoplasms. More than 90% of ovarian cancers arise from epithelial cells (6).

Fertility drugs, androgens, and other hormones used in replacement therapies are widely recognized as risk factors for gynecological cancers (7, 8). Therefore, it has been proposed that endocrine hormones are critical to the development of malignant gynecological neoplasms. Hormone receptors are classified into three superfamilies: GPCRs, cytokine receptors, and nuclear receptors. Generally, water-soluble hormones bind GPCRs and cytokine receptors located on the cell membrane surface, triggering a cascade of signaling events. Lipid-soluble hormones enter the cell and bind to nuclear receptors that can directly regulate gene transcription. The role of the nuclear and cytokine receptor families in ovarian cancer has been well established. For example, estrogen has been implicated in the progression of ovarian cancer, where estrogen transduces pro-metastatic pathways *via* the nuclear estrogen receptor (ER). Recent epidemiological studies have demonstrated an elevation of ovarian cancer incidence with the postmenopausal use of estrogen (7, 9).

G protein-coupled receptors are involved in many aspects of tumorigenesis, including the promotion of aberrant growth, increased cell viability, angiogenesis, and metastasis (10). Recent large-scale genomic analyses have discovered an abundance of mutations in G proteins and GPCRs (11). For instance, 20% of all sequenced human tumors contain mutations in GPCRs, including mutations in the thyroid-stimulating hormone receptor (TSHR) (12), the luteinizing hormone receptor (LHR), and the follicle-stimulating hormone receptor (FSHR), known to be involved in thyroid, breast, lung, and colon cancers, respectively (13). In another study, a mutant allele of GPRC5A was found to affect breast cancer risk (14), as lower levels of GPRC5A had adverse effect on the expression and function of *BRCA1*. Alterations in gene expression and promoter methylation of GPCRs in tumors have also been reported (10), and these changes appear to promote cancer proliferation, immune evasion, invasion of surrounding tissues, and increased resistance to hostile environments, such as hypoxia (15). In a study describing glioblastoma GPCR transcriptomes, 138 GPCRs were found to be aberrantly expressed, including several orphan receptors, such as GPR19, GPR82, GPR171, and GPR128 (16). Moreover, the orphan receptor GPR161 was shown to be overexpressed in triple-negative breast cancer and correlated with poor prognosis (17). In the same study, GPR161 was found to be a major regulator of cell proliferation and migration through induction of rapamycin signaling.

Here, we will review the role of endocrine GPCRs in ovarian cancer. The term “endocrine GPCRs” refers to a subgroup of GPCRs with endogenous ligands (i.e., it excludes the G-protein-coupled olfactory receptor). This review will focus on the following: (1) reproductive hormone receptors, including the G protein-coupled estrogen receptor (GPER), FSHR, and LHR; (2) hormone receptors that are involved in gonadotropin release, including the kisspeptin receptor (Kiss1R) and gonadotropin-releasing hormone receptor (GnRHR); (3) other hormone receptors including endothelin receptors (ETRs) and angiotensin II type 1 receptor (AGTR1). The signaling network in different receptors is also described, and perspectives on the future of GPCR research in ovarian cancer research are given.

## G Protein-Coupled Estrogen Receptor

Estrogens are sex hormones involved in regulating cell growth and differentiation in mammalian ovaries. A large-scale prospective cohort study conducted in the United States showed a strong link between ovarian cancer and estrogen. An increase in the risk of ovarian cancer was found in patients who had undergone estrogen replacement therapy (ERT), with the rate ratio (RR) rising to 1.23 [95% confidence interval (CI), 1.06–1.43]. For patients who had undergone ERT for more than 10 years, the RR increased to 2.2 (95% CI, 1.53–3.17) (7). This prospective study provided strong evidence that estrogen is a high-risk factor in postmenopausal women. However, a meta-analysis of 15 case studies did not find any correlation between ERT and ovarian cancer. The apparent contrast in results between each study may be due to the different genetic backgrounds of the patients tested or the sample sizes of each study. It is worth noting that ERT consists of treatment with both estrogen and another sex hormone, progesterone. These two hormones have opposing effects on ovarian epithelial cells. Therefore, the effects of estrogen and progesterone should be considered and investigated separately.

Estrogens bind to two different types of receptors in mammalian cells. Two well-known ERs, ERα and ERβ, are nuclear receptors that affect gene expression by binding to estrogen responsive elements in the promoter region of target genes. Another is GPER (also known as GPR30), which belongs to the GPCR family of receptors, and mediates the non-genomic signaling of estrogens. GPER is expressed in various cancer cell lines and primary tumors of the breast, endometrium, ovaries, thyroid, lung, prostate, testicular germ cells, and brain (18). Even though GPER is widely expressed in tumors, its role in ovarian cancer is controversial. An early report proposed that elevated expression levels of GPER correlate with poor prognosis (19). Activation of GPER in ER-negative cells has also been shown to promote cell migration and invasion (20). However, contradictory results suggesting that high GPER expression is associated with a favorable prognosis have also been published (21, 22). In fact, the GPER selective agonist, G-1, significantly inhibited the proliferation of ovarian cancer cells by suppressing tubulin polymerization and arresting cell cycle progression (2, 23). These conflicting results indicate that the role of GPER in ovarian cancer may vary from case to case. Several recent studies have suggested that cross talk between GPCRs might clarify the role of GPER during tumorigenesis. For example, the ability of GPER to serve as a prognosticator of cancer prognosis depends on gonadotropin receptor status. Increased expression of GPER predicted a longer survival period in patients negative for either FSHR or LHR compared to patients who were positive for FSHR and LHR. In addition, patients who tested negative for both FSHR and LHR had a more favorable prognosis than single-receptor negative patients (21). This observation suggests a mutually exclusive effect of GPER in association with LHR and FSHR during ovarian cancer progression.

With regards to the mechanistic activity of GPER, it appears that a signal transduction occurs primarily through the extracellular signal-regulated and mitogen-activated protein kinase pathway. It has been shown that activation of GPER by estradiol induces ERK1/2 phosphorylation and promotes ovarian cancer proliferation regardless of ER status (24). In addition, GPER also activates cAMP and PIP2 signaling, inducing expression of matrix metalloproteinase 2 (MMP2) and MMP9, which, in turn, promote cancer metastasis (25). In breast and thyroid cancer cells, as well as endometrial cells, GPER exerts its effects through the transactivation of the epidermal growth factor receptor (EGFR). For example, GPER activates the ERK1/2 pathway *via* the transactivation of EGFR in breast cancer cell lines (26). In ovarian cancer cells, activation of GPER promotes cell survival *via* the transactivation of EGFR and cross talk with the PI3K/AKT signaling pathway (27).

The expression level of GPER is tightly associated with cell survival in epithelial ovarian cancer cells; a higher expression of GPER is correlated with a lower survival rate. Long et al. found that GPER expression correlates with tumor size and stage, lymph metastasis, and MMP9 expression (20, 28). ER-negative cells provide a clear picture of the role of GPER in ovarian cancer cell proliferation. 17β-Estradiol is a strong agonist of GPER that can enhance S-phase promotion and cell migration in ER-negative ovarian cancer cells (19, 20). Selective activation of GPER by G-1 can also activate EGFR, upregulate c-fos, cyclin D1, cyclin E, and cyclin A, and promote cell proliferation (23). These *in vitro* studies provide strong evidence that GPER promotes ovarian cancer cell proliferation. However, a clinical study involving 40 ovarian cancer patients with higher GPER expression found no association between clinical stage, pathological stage, and survival time (29). Tissue specimens from 124 ovarian cancers, 35 benign tumors, and 35 low-malignant tumors revealed that GPER is downregulated in ovarian cancer and that elevated expression of GPER correlates with a longer survival time. Additionally, GPER overexpression can induce G2/M cell cycle arrest *via* cyclin B1 and CDC2 (22). Therefore, the ultimate result of GPER activation varies when co-expressed with other hormone receptors. For example, the effect of GPER activation is not only regulated by FSHR and LHR but also ER. It is apparent then that the role of GPER during ovarian cancer progression is highly complex and requires further investigation.

## Gonadotropin-Releasing Hormone Receptor

Two forms of GnRHR have been discovered in mammals: GnRHR1 and GnRHR2. GnRHR1 is predominantly expressed in the hypothalamus and the pituitary and regulates reproduction in response to gonadotropin-releasing hormone (30). GnRHR2 is mainly expressed in the midbrain. GnRHR2 appears to be involved in the regulation of sexual behavior and food intake (30). However, human GnRHR2 acquires frame shift mutations resulting in the appearance of an early stop codon and the production of a truncated version of the protein. Therefore, in this review, we will primarily focus on GnRHR1, hereby referred to as GnRHR.

Upon activation by its ligand (GnRH), GnRHR stimulates the synthesis and release of the gonadotropic hormones, FSH and LH. Because GnRHR is expressed in the ovary (31, 32), it is widely believed to be involved in ovarian cancer development and metastasis. The potential role of GnRHR as a tumor suppressor in ovarian cancer has been hypothesized because ovarian cancer patients with lower tumor expression levels of GnRHR showed more favorable survival rates (31). *In vitro* experiments also support this relationship as treatment with a specific GnRHR agonist (Buserelin) inhibited phosphatidylinositol kinase and exhibited a strong anti-mitogenic effect on ovarian carcinoma cells (33). In another study, the GnRHR agonist, [d-Ala6] GnRH, directly inhibited the growth of ovarian cancer cells in a time- and dose-dependent manner, whereas a GnRHR antagonist (Antide) reversed this effect (34).

Estradiol (E2) downregulated the expression of GnRHR and reduced GnRHR-mediated inhibition of proliferation in the ovarian cancer cell line, OVCAR-3. However, in human ovary surface epithelial (hOSE) cells, estradiol did not affect the expression of GnRHR, and thus GnRH did not affect cell proliferation (35). These results indicate that GnRHR expression in ovarian cancer can be suppressed by the estrogen signaling pathway. Another study found that estrogen repressed GnRHR-mediated inhibition of proliferation in an ERα dependent manner *via* the upregulation of c-Jun and the recruitment of the cAMP response element binding (CREB) protein (36). These results provide evidence that estradiol promotes the proliferation of ovarian cancer cells and overrides the antineoplastic effects of GnRHR. Because the role of GPER in OVACR-3 and hOSE cell proliferation and migration is unclear, further studies are required to explore the potential cross talk between GnRHR and GPER in regulating ovarian cancer progression.

The signaling pathways regulated by GnRHR appear to be tissue specific. Classic GnRHR signaling in pituitary gonadotrophs is responsible for the activation of protein kinase C (PKC), phospholipase C (PLC), and adenylyl cyclase. Upon ligand binding, GnRHR activates phosphotyrosine phosphatase (PTP), which inactivates the epidermal growth factor receptor/mitogen-activated protein kinase signaling pathway *via* dephosphorylation of the EGFR. GnRHR inhibits proliferation through the activation of PKC, which is followed by phosphorylation and activation of ERK1/2. Interestingly, this antiproliferative effect can be reversed by blocking GnRHR, which has been shown to play a critical role in this process. With respect to GnRHR2, it has been reported that this receptor is non-functional (37). However, the role of GnRHR2 in ovarian cancer requires further investigation. One study showed that treatment with GnRH-I or GnRH-II inhibits PTP and subsequently inactivates MAPK signaling. More specifically, analogs of GnRH-I and GnRH-II reduced EGF-triggered mitogenic signal transduction (38). Transcriptomic and proteomic approaches were used to investigate the effects of triptorelin, an agonist of GnRHR, on ovarian cancer cells. Triptorelin has been shown to be an effective promoter of cell cycle arrest by inhibiting G2/M phase progression, eventually leading to apoptosis. This effect appears to be mediated by NF-κB phosphorylation and AKT inactivation (39).

In contrast to the studies listed above, it has been shown that GnRHR antagonists exhibit antiproliferative properties in ovarian cancer cells by inducing cell apoptosis *via* the activation of p38 and c-Jun mediated Bax expression. Increased Bax expression ultimately leads to mitochondrial dysfunction and subsequent activation of the intrinsic pathway of apoptosis (40). Intriguingly, GnRHR antagonists appear to be less cytotoxic and more potent in mouse models of cervical cancer compared to GnRHR agonists (41). The uniformity of the GnRH-II agonist and antagonist on cancer cell proliferation probably result from cell stress caused by hyperactive or over deterrence related signaling. Cross talk between EGF and GnRH-II signaling also appears to promote ovarian cancer metastasis. Specifically, EGF induces the expression of GnRH-II by promoting CREB-dependent transcription, resulting in ovarian cancer invasion (42). Other studies have shown that GnRH-II can enhance cancer cell adhesion, thereby promoting migration and invasion, by increasing laminin receptor expression levels (43).

In conclusion, GnRHR is a critical regulator of ovarian cancer cell proliferation. Both hyperactivity (i.e., high doses of GnRH, ~100 nM) and inhibition of GnRHR can suppress cancer cell proliferation and induce apoptosis. However, lower doses of GnRH-II (~10 nM) can promote cancer cell invasion and migration. Therefore, imbalances in the levels of GnRHR activity appear to modulate the rate of cancer cell proliferation and metastasis.

## Follicle-Stimulating Hormone Receptor

All ovarian epithelial tumors express FSHR, and the expression level of FSHR is positively correlated with tumor grade (44). FSH is known to stimulate ovarian cancer cell proliferation, and this effect can be reversed by exposure to LH. These observations may explain why FSH treatment does not increase ovarian cancer risk in postmenopausal women (45). LHR and FSHR are generally co-expressed in the ovaries of postmenopausal women, and the co-regulation of LHR and FSHR signaling is essential to maintain normal function of the ovaries. Investigation into the underlying molecular pathogenesis of ovarian cancer has provided evidence that FSHR activation can influence cancer related gene expression. For example, FSH can downregulate tumor suppressor genes, including RB1 and BRCA1 (46), and overexpression of FSHR increased protein levels of Her2, c-myc, EGFR, and ERK1/2 (47), resulting in ovarian cancer cell proliferation. In addition, FSH was found to regulate ovarian cancer mitosis *via* the PI3K/AKT/HIF-1/cyclin-D1 signaling pathway (48). It appears that FSHR not only enhances cell growth but also promotes the invasiveness of ovarian cancer cells. Furthermore, it has been shown that activation of FSHR triggers the PI3K/AKT/Snail signaling pathway, activates ERK1/2, and upregulates expression of OCT4, thereby promoting cancer cell epithelial–mesenchymal transition, migration, and distant invasion (49, 50).

Several groups have already begun testing FSHR inhibitors for their ability to inhibit the progression of ovarian cancer. In one study, an FSH analog complexed with either paclitaxel or cisplatin inside nanoparticles enhanced the potency and selectivity of the chemotherapeutic drug to target ovarian cancer cells, while showing a reduction of unwanted side effects. Here, the selectivity of these complexed nanoparticles for ovarian cancer cells was enhanced using an FSH peptide conjugated to poly-amidoamine dendrimers. These particles have been previously shown to perform better than non-targeted administration with regards to inhibition of ovarian cancer proliferation and lymphatic metastasis (51, 52). Moreover, FSHR-based targeting has shown to have potent anticancer effects in both *in vitro* and *in vivo* models (53). In another study, anti-FSHR immune receptors were used to redirect T cells to ovarian tumors by inducing the expression of anti-FSHR immune receptors in the patient’s T lymphocytes. The anti-FSHR immune receptor then triggered T-mediated cytotoxicity in ovarian cancer cells (54). This promising approach could potentially recruit T lymphocytes to ovarian cancer tumors and not only initiate T cytotoxicity and continuous tumor immune responses but also reduce adverse side effects by limiting cytotoxicity to the site of the tumor.

## Luteinizing Hormone Receptor

Previous studies have shown LHR to be expressed abundantly in the plasma membrane of ovarian epithelial cells (55). Moreover, low-grade ovarian cancer tumors express LHR to a higher degree than high-grade tumors, which suggests that LHR may play a role in ovarian cancer progression. LH is known to regulate the expression of several genes related to cell growth and apoptosis. It is worth noting that LH generally upregulates genes related to proliferation. Studies have shown that LH induces the expression of ERBB receptor tyrosine kinase 2 (ERBB2), which promotes cell proliferation. However, upregulation of ERBB2 alone was insufficient to enhance cell proliferation and survival of ovarian cancer cells (56). Interestingly, it was also found that activation of the LHR reduces cell invasion and proliferation. Studies have shown that MMP family members, such as MMP2 and MMP9, are downregulated by LHR activation, while cell adhesion and basement membrane proteins (COL4A3, COL4A4, NID2, ITGB8, and LAMA3) were upregulated (56). These results might explain why treatment with LH suppresses ovarian cancer invasion. By screening using an miRNA-specific array, several antiproliferation miRNAs, including miR-101, miR-301, and miR-210, were found to be upregulated by LH treatment (57). Furthermore, LH appears to activate the PTP pathway *via* Ga(i) and counteracts mitogenic signal transduction induced by EGF (58), suggesting that the interaction of LHR and EGFR is essential for determining the fate of ovarian cancer cells.

Because LHR localizes to the cell surface of ovarian cancer cells, several studies have used LHR as a cancer biomarker for targeted therapy and have obtained moderately positive results. For example, using a nanoparticle that links CD44-siRNA to an LH analog in combination with paclitaxel significantly enhanced cell death in ovarian tumors (59). However, as LHR expression is generally decreased during tumorigenesis, this treatment would not be suitable for advanced stages of ovarian cancer.

## Thyroid-Stimulating Hormone Receptor

Because TSHR is mainly found in the thyroid, scientists have primarily focused on the role of TSHR in thyroid cancer progression. However, recent evidence suggests that TSHR is highly expressed in ovarian cancer tumors (60). Patients with high tumor TSHR expression levels had lower survival rates compared to patients with low TSHR tumor expression levels (61). There remain discrepancies about the role of TSHR in thyroid and ovarian cancers: (1) TSHR was found to be downregulated in thyroid cancer but highly expressed in ovarian cancer and (2) high TSHR expression was found to predict increased survival rates for thyroid cancer patients but poorer outcomes for patients with ovarian cancer (62). Therefore, TSHR activity appears to be correlated to ovarian cancer progression. However, further investigation is required to discern the role of TSHR in ovarian tumorigenesis.

## Kisspeptin Receptor

Kisspeptin receptor has been identified as a tumor suppressor in breast cancer and melanoma (63, 64). However, recent evidence suggests that kisspeptin (Kiss1) and Kiss1R are involved in ovarian cancer progression. Despite the similarity of Kiss1R expressions levels between malignant and benign ovarian tumors, the expression of Kiss1 was found to be significantly higher in malignant ovarian tumors. More importantly, Kiss1 expression levels negatively correlated with the clinical stage diagnosis (65). Immunohistochemical analysis of 518 ovarian cancer tumor samples suggested a favorable prognostic role of Kiss1R with regards to the total survival duration as well as the disease-free survival period. Therefore, it appears that the activation of Kiss1/Kiss1R indicates a favorable prognosis. Patient plasma kisspeptin levels also correlated with cancer metastasis, and levels of kisspeptin lower than 20 pmol/L have been associated with a higher risk of ovarian cancer metastasis (66). Additionally, high expression levels of Kiss1R decreased the lysophosphatidic acid induced migration of ovarian cancer cells. In another study, kisspeptin treatment inhibited cancer cell migration in a PKC-dependent manner (67), and decreased stromal cell-derived factor 1 (SDF-1) mediated tumor migration by suppressing AKT phosphorylation (63). Kisspeptin-10 can also activate Kiss1R and influence the binding efficiency of SDF-1/CXCL12 with its cell surface receptor, CXCR4, thereby inhibiting metastasis. Altogether, these results are indicative of the tumor suppressing nature of Kiss1 and its receptor, Kiss1R, in ovarian cancer.

## Angiotensin II Type 1 Receptor

Angiotensin II type 1 receptor is a key member of the renin–angiotensin system. This receptor is mainly expressed in the liver, lungs, kidneys, and adrenal glands. Recently, a study demonstrated, by immunohistohemical staining, that ovarian tumors express AGTR1. Survival analysis (from The Cancer Genome Atlas database) found that the expression level of AGTR1 is directly related to the overall survival and disease-free survival rate and that high AGTR1 expression indicates an unfavorable prognosis. Pathologic analysis showed that elevated expression of AGTR1 correlated with high microvessel density and an increased secretion of vascular endothelial growth factor (VEGF) (68). Serum levels of angiotensin II converting enzyme (ACE), the key enzyme responsible for production of angiotensin II, were shown to be significantly increased in ovarian cancer patients. However, no correlation was found between angiotensin II and Ca-125, a glycoprotein biomarker of advanced ovarian cancer. These results suggest that changes in the expression levels of ACE are an early event during carcinogenesis and could be used as an efficient biomarker for the diagnosis of ovarian cancer compared to angiotensin II or Ca-125. Because ovarian cancer cells invade surrounding tissues through the peritoneal cavity, understanding the role of angiotensin II and ACE during metastasis will be essential to assess how AGTR1 regulates cancer progression. Future studies will determine the correlation between serum and peritoneal fluid angiotensin II levels and ovarian tumorigenesis.

BRCA1, one of the most well-studied tumor suppressor genes, is responsible for DNA repair post injury. Studies have shown that there is a positive correlation between BRCA1 and AGTR1 levels in ovarian tumors. Wild-type BRCA1 patients show higher tumor expression levels of AGTR1 compared to patients with BRCA1 mutations. Elevated expression of BRCA1 has been shown to significantly promote the expression of AGTR1. However, knockdown of BRCA1 did not affect AGTR1 expression. The positive regulation of AGTR1 by BRCA1 implies that AGTR1 might maintain the integrity of the cell’s genome and reduce apoptotic signaling throughout carcinogenesis, without promoting cell mitosis (69). Other studies have demonstrated that angiotensin II can increase endothelial nitric oxide (eNOS) and upregulate cyclooxygenase-2, thereby enhancing angiogenesis (70). In mesenchymal stem cells, AGTR1 induced HIF-1a expression and resulted in the upregulation of VEGF and ACE. In general, VEGF stimulates endothelial cell proliferation and migration, processes which contribute to tumor angiogenesis, while ACE accelerates the *de novo* production of angiotensin II, which forms a positive feedback loop (71). Therefore, AGTR1 antagonists might be useful for suppressing tumor angiogenesis in ovarian cancer. Targeting AGTR1 could significantly inhibit tumor growth *via* inactivation of the phosphorylation of PLC β3, which could disrupt tumor angiogenesis by reducing the VEGF production, thus inhibiting endothelial cell survival (72). However, the source of the VEGF secretion (either endothelial cells or ovarian cancer cells) and the molecular mechanism responsible for angiotensin II-mediated endothelial cell migration and microvessel formation remain unknown. Thus, elucidating the role of AGTR1 during ovarian cancer metastasis will prove integral to developing AGTR1-targeted drugs for the treatment of ovarian cancer.

## Endothelin Receptor

There exist two types of ETRs: the endothelin A receptor (ETAR) and the endothelin B receptor (ETBR). All metastatic ovarian cancers and 90% of primary ovarian cancers express ETAR, whereas around 40% of ovarian tumors express ETBR. The ovarian cancer cell lines, PEO4 and PEO14, not only express ET-1 (endothelin 1) and ET-3 (endothelin 3) but also ETAR and ETBR, which suggests that ovarian cancer cells stimulate the ETR pathway in an autocrine fashion. ETAR agonists have been shown to exhibit strong antitumor activities compared to ETBR agonists, indicating that ETAR plays a more significant role during cancer progression in response to endothelin exposure compared to ETBR (73). One study found that endothelin could induce VEGF production *via* the HIF-1a pathway, resulting in neovascularization of ovarian tumors (74). Endothelin also induced cyclooxygenase 1/2 expression and increased expression levels of prostaglandin E2 and prostaglandin E4, resulting in VEGF production and promotion of angiogenesis. Furthermore, the activation of ETRs upregulated MMP-2, -3, -7, -9, and -13, critical mediators of cellular invasion. Integrin-linked kinase (ILK) also appears to be involved in transducing extracellular endothelin signals, mediating the activation of PI3K/AKT and promoting cell motility and invasion (74). It was also found that β-arrestin acts as an ETAR signal transducer by inactivating GSK-3β through the PI3K/ILK/AKT pathway, thereby promoting WNT signaling and contributing to the chemoresistance, invasion, and metastasis of ovarian tumors.

Drug resistance is a major challenge in the treatment of ovarian cancer with paclitaxel therapy. Because endothelial cells generally enhance ovarian cancer cell survival, previous attempts to target ovarian tumors included the use of an ETR agonist (Atrasentan, ABT-627) to inhibit cell proliferation and VEGF production and to reduce ILK expression and phosphorylation of GSK-3β (75). Ovarian tumors are known to become resistant to paclitaxel. However, administration of ABT-627 in combination with paclitaxel yielded improved results with regards to the inhibition of angiogenesis and the induction of apoptosis. In a separate study, the endothelial receptor antagonist, ZD4054, reduced cancer cell survival, invasion and angiogenesis and enhanced the chemotherapeutic sensitivity of ovarian tumors. ZD4054 effectively induced apoptosis by inhibiting Bcl-2 and activating caspase 3 (76). Furthermore, it was found that gene silencing of the endothelial converting enzyme resulted in the suppression of endothelial mediated cell proliferation and invasion *via* inhibition of MAPK phosphorylation, which suppressed MMP2 activity and upregulated the ratio of E-cadherin to N-cadherin (77). Finally, epigallocatechin-3-gallate, a polyphenol derived from green tea considered to be a potent natural anticancer small molecule, has been shown to reduce ovarian cancer progression by targeting ETAR, making it a potentially cost effective method of preventing ovarian cancer.

## The Receptor Network in Ovarian Cancer

The physiology of the ovary is precisely regulated by the reproductive hormones and receptors of the HPG axis. These receptor signaling cascades are not only found in the ovary but also in ovarian tumors. Evidence suggests that ovarian cancer development is tightly associated with dysregulated hormonal signaling. More importantly, hormone receptor expression patterns and the modulation of intricate signaling networks appear to determine the fate of cancer cells. The role of GPCRs in regulating ovarian cancer development is summarized in Table 1. In our model, Kiss1R, GnRHR, and LHR act as cancer suppressors by inhibiting proliferation and metastasis. FSHR acts as an oncogene in ovarian cancer by promoting cell proliferation and survival and antagonizes the tumor suppressing effects of LHR. GPCRs involved in cardiovascular function, such as AGTR1 and ETR, can also modulate the progression of ovarian cancer. Activation of AGTR1 and ETR has been shown to enhance angiogenesis and promote metastasis. While the effects of AGTR1 and ETR activity during tumorigenesis have been well studied, the relationship between these receptors and classic reproductive hormone GPCRs requires further investigation. In conclusion, numerous GPCRs and their specific ligands are likely important in ovarian cancer development and metastasis. Therefore, fully characterizing the roles of these GPCRs during tumorigenesis will greatly benefit the development of novel chemotherapeutics.

**Table 1:** **Summary of the role and pathways of G-protein-coupled receptors in ovarian cancer**.

Receptor	Ligand	Function	Pathway	Reference
G-protein-coupled estrogen receptor	Estradiol	Regulate metastasis, and proliferation	PI3K/AKT/MMP-9	(24–27)
GnRHR1	GnRH-I, GnRH-II	Antiproliferation	P38/c-Jun/MAPK, Bax/caspase9	(37–39)
Follicle-stimulating hormone receptor	Follicle-stimulating hormone	Enhance proliferation, cell survival, metastasis	PI3K/AKT/HIF-1/cyclin-D1, ERK1/2MAPK	(47–50)
Luteinizing hormone receptor	Luteinizing hormone	Inhibits metastasis, suppress proliferation	cAMP/phosphotyrosine phosphatase/EGFR	(56, 58)
Thyroid-stimulating hormone receptor	Thyrotropin	Predicate good outcome	Unknown	(61, 62)
Angiotensin II type 1 receptor	Angiotensin II	Enhance angiogenesis	eNOS/cyclooxygenase-2 (COX2)/HIF-1α/vascular endothelial growth factor (VEGF)	(68, 70, 71)
Kisspeptin receptor	Kisspeptin	Inhibits proliferation and migration	Stromal cell-derived factor 1/CXCL12/CXCR4/AKT	(63, 67)
Endothelin receptor	Endothelin	Enhance angiogenesis and metastasis	PI3K/integrin-linked kinase/AKT, COX2/PE/HIF-1α/VEGF	(74–77)

## Author Contributions

All authors have made substantial, direct, and intellectual contribution to the work and approved it for publication.

## Conflict of Interest Statement

The authors declare that the research was conducted in the absence of any commercial or financial relationships that could be construed as a potential conflict of interest.
